# Hyaluronan, a double-edged sword in kidney diseases

**DOI:** 10.1007/s00467-021-05113-9

**Published:** 2021-05-19

**Authors:** Aditya Kaul, Kavya L. Singampalli, Umang M. Parikh, Ling Yu, Sundeep G. Keswani, Xinyi Wang

**Affiliations:** 1 Laboratory for Regenerative Tissue Repair, Division of Pediatric Surgery, Department of Surgery, Texas Children’s Hospital/Baylor College of Medicine, Houston, TX 77030 USA; 2 Medical Scientist Training Program, Baylor College of Medicine, Houston, 77030 TX USA; 3 Department of Bioengineering, Rice University, Houston, 77030 TX USA

**Keywords:** Acute kidney injury (AKI), Chronic kidney disease (CKD), Diabetic nephropathy, Hyaluronic acid (HA), IgA nephropathy, Kidney cancer, Kidney fibrosis, Obstructive uropathy, Vesicoureteral reflux (VUR)

## Abstract

Over the years, hyaluronic acid (HA) has emerged as an important molecule in nephrological and urological studies involving extracellular matrix (ECM) organization, inflammation, tissue regeneration, and viral sensing. During this time, many have noted the perplexing double-edged nature of the molecule, at times promoting pro-fibrotic events and at other times promoting anti-fibrotic events. Different molecular weights of HA can be attributed to these disparities, though most studies have yet to focus on this subtlety. With regard to the kidney, HA is induced in the initial response phase of injury and is subsequently decreased during disease progression of AKI, CKD, and diabetic nephropathy. These and other kidney diseases force patients, particularly pediatric patients, to face dialysis, surgical procedures, and ultimately, transplant. To summarize the current literature for researchers and pediatric nephrologists, this review aims to expound HA and elucidate its paradoxical effects in multiple kidney diseases using studies that emphasize HA molecular weight when available.

## Introduction

Although the origin of kidney injuries can be diverse, most lead to kidney failure, largely characterized by fibrosis [1]. Between 1995 and 2010 in the USA alone, pediatric stage 5 chronic kidney disease (CKD 5) grew in incidence from 14.6 to 15.7 cases per million population and in prevalence from 71 to 89 cases per million population. Together, these numbers present a major healthcare and psychosocial burden for young patients and their families [2]. Deciphering how kidney injuries promote fibrogenesis is critical for developing innovative therapies to prevent kidney failure.

Hyaluronan (HA), a glycosaminoglycan ubiquitously expressed by most vertebrates, has a versatile structure and function and has been increasingly implicated in the pathogenesis of fibrosis. On one hand, HA has been shown to mediate inflammatory and pro-fibrotic reactions [3]; on the other hand, high molecular weight-HA (HMW-HA) variants can exert anti-inflammatory and anti-fibrotic effects [4, 5]. Therefore, understanding how distinct molecular weight (MW) variants of HA transduce signals that lead to either regenerative or fibrotic responses can help combat the clinical and economic burdens of kidney disease.

The following sections will cover the current understanding of HA in kidney-related diseases while discussing its pathophysiological roles as either a mediator or cytoprotector of these diseases.

## Hyaluronic acid and its biological and physiological properties

Hyaluronan (HA) is a non-sulfated glycosaminoglycan (GAG) composed of linearly repeating [glucuronate- β 1,3-N-acetylglucosamine- β 1,4-] residues without a core protein [6]. Each disaccharide unit of the HA chain contains one carboxyl group, giving the poly-disaccharide an overall negative charge and enabling it to act as a water reservoir within the extracellular matrix (ECM) to provide hydration and structural and mechanical support [7]. While most research highlights the relevance of HA quantity in the context of pathological consequences, few have considered the importance of HA molecular mass and structure [8].

### Hyaluronan synthesis, degradation and inhibition

Despite its simple core structure, the biological processes of HA synthesis and degradation are tightly controlled to mediate its structural and biological functions. HA synthases (HAS) produce HA whereas hyaluronidases (HYAL) degrade HA, both working in concert to regulate baseline HA levels and HA molecular weight. Historically, high molecular weight (HMW)-HA (> 2000 kDa) was thought to be exclusively synthesized by *HAS1* and *2*, with low molecular weight (LMW)-HA being mainly produced by *HAS3* [9]. However, Itano et al. reported that all three *Has* enzymes contribute to HMW-HA synthesis, with *Has2* specifically generating a larger HA variant above 2000 kDa [10].

Functionally, HAS 1–3 proteins exhibit a unique expression profile during development. One group examined all three HA synthases through the stages of murine embryogenesis and showed that each synthase is differentially expressed with respect to organ, timepoint, and concentration (Table 1) [11]. As observed here, *Has1–3* are expressed at embryo (E) day 9, but the *Has2* knockout mouse displays embryonic lethality. In contrast, *Has1* and *Has3* knockouts and *Has1/3* double knockouts do not show any such abnormality [12]. These studies suggest a pivotal role for HAS2 in embryogenesis.

**Table 1:** HAS* 1–3*** gene expression pattern during murine embryonic development**. (“Plus” signs indicate relative concentration of each molecule.) All three *Has* synthases are present in the indicated organs at various timepoints throughout embryo development. Major takeaways include the highest expression of all three *Has* synthases in the heart at E9, with skin showing the highest expression relative to other organs from E11 to at least E17. At E17, *HAS1–3* are observed in the kidney and vitreous body for the first time

	E9 (embryonic stage)			E11-13		E15		E17			
	Neural tube	Lung	Heart	Dermis	Mesenchyme	Epidermis	Cartilage	Epidermis	Bone	Kidney	Vitreous body
*Has1*	**++**	**++**	**+++**	**+++**	**++**	**++**	**++**	**++**		**++**	**+**
*Has2*	**+++**	**+++**	**+++**	**+++**	**+**	**++**	**+++**	**+++**	**++**	**++**	**+**
*Has3*	**+**	**+**	**+++**	**+++**	**++**	**++**	**++**	**+++**	**++**	**++**	**+**

HAS1 synthesizes less HA than HAS2 and HAS3 physiologically, but its activity surges upon pathological events. In support of these observations, HAS1 functions have been summarized as follows: (1) *Has1* synthesized HA attracts more leukocytes; (2) *Has1* activation is preferentially induced by inflammatory reactions; and (3) both full-length and alternatively spliced HAS1 protein isoforms have been observed in cancers [13]. Further research is needed to establish the distinct functions of the HA synthases, especially in the context of injury or disease.

Catabolic HA enzymes, specifically HYALs 1 and 2, cleave HMW-HA into LMW-HA and oligosaccharides [14, 15], with deficiency of these two HYALs leading to malfunction of organs like the kidney [16], heart [17], and lungs [18]. Conversely, HA accumulation has not been observed in *Hyal3* knockout mice [15], suggesting that *Hyals 1* and *2* may compensate for the loss of *Hyal3*. In the next section, we will discuss the paradoxical functions of HA that arise due to its MW variations and complex binding mechanisms.

## How molecular weight relates to HA function

The double-edged sword approach of this review arises from the fact that HA can influence both pro- and anti-inflammatory/fibrotic reactions. Studies have reconciled this paradox by classifying HA as either pro-inflammatory/fibrotic LMW-HA or anti- inflammatory/fibrotic HMW-HA [10, 19, 20]. Per the literature, oligosaccharides are 1.7–6.1 kDa in size [21], LMW-HA is < 120 kDa in size [22], and HMW-HA is > 900 kDa [23].

HMW-HA (> 900 kDa) is the main variant observed in healthy solid tissue across species [24], and HMW-HA increases in response to body injury. We have shown that HMW-HA synthesis can be upregulated by IL-10 and it can improve regenerative repair in multiple organs [19, 22]. HMW-HA has also been shown to improve diabetic wound healing and restore skin integrity typically lost to fibrosis in animal models [25]. In contrast, LMW-HA typically creates a pro-inflammatory state, which can initially be beneficial as it activates an immune response to remove pathogens and prevent infection after injury [26]. However, the persistence of LMW-HA in disease states leads to tissue damage and poor remodeling.

**Fig. 1:**
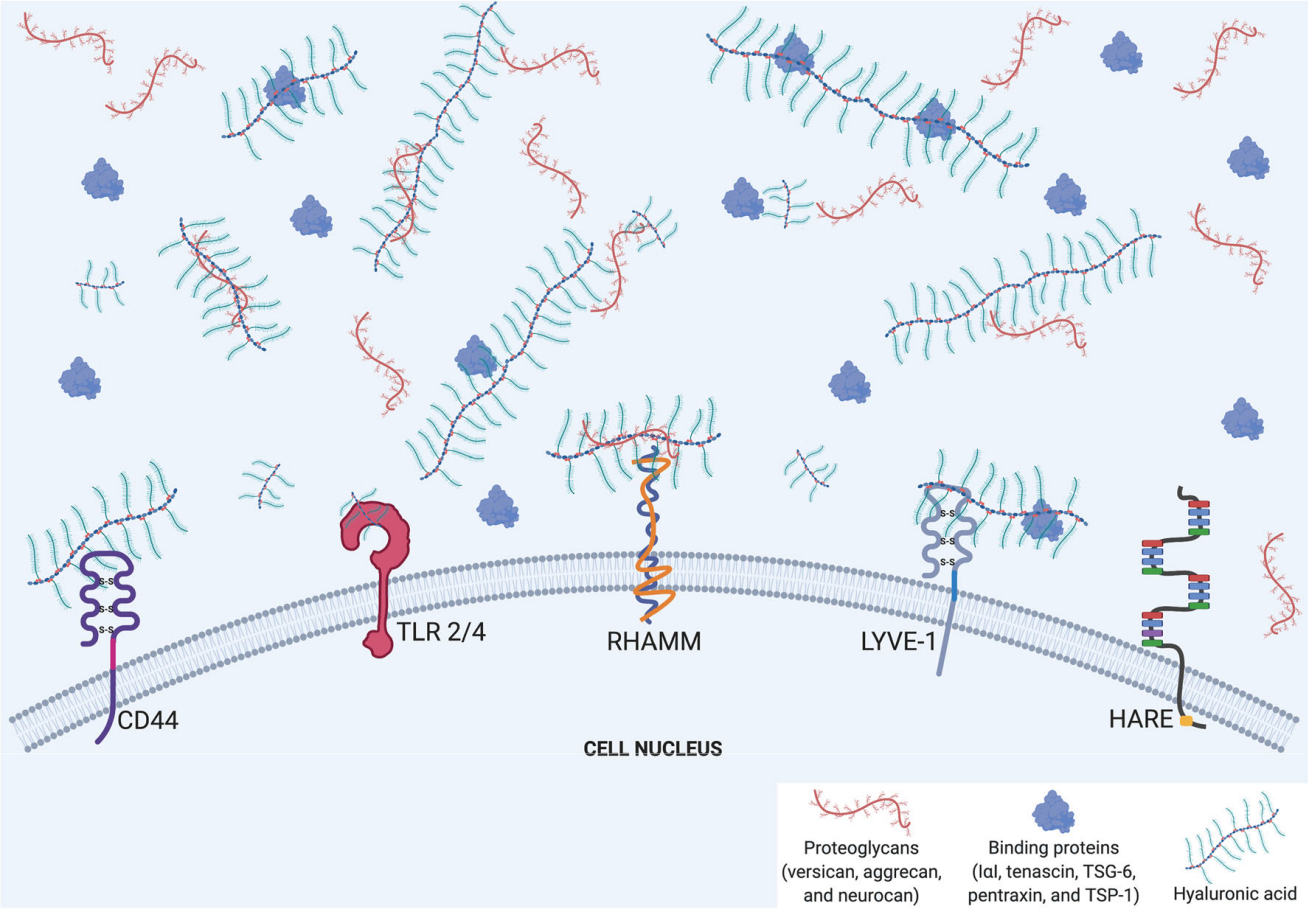
**Schematic illustration of the interactions of extracellular hyaluronan (HA) and its receptors and binding proteins.** This figure illustrates the common binding mechanisms for extracellular HA. The major HA receptors include CD44 and its homolog, LYVE-1, in addition to TLR 2/4, RHAMM, and HARE. These receptors are present at the cell surface, with extracellular HA binding with or without binding proteins (specified in the legend). The fact that HA and its binding proteins are located outside the cell indicates that HA is a major component of pericellular coats. The interaction between HA and its receptors can be preferential, as LMW-HA tends to bind TLRs 2/4, although some, like CD44, bind both LMW- and HMW-HA. The depiction of different sizes of HA corresponds to LMW and HMW variants, which are responsible for the dichotomous pro-inflammatory/pro-fibrotic effects to anti-inflammatory/anti-fibrotic effects, respectively. In addition, crosslinks between HA and various binding proteins, including proteoglycans and other hyaladherins, modifies HA’s effects. This schematic is a broad illustration and is not drawn to scale. Moreover, that five receptors are shown on one cell surface is for illustrative purposes only. Created with BioRender.com

Based on research in other organs, we expect changes in HA distribution from HMW to LMW to be associated with kidney disease. Consistently, our mouse unilateral ureteral obstruction (UUO) model shows increased HMW-HA at early time points after kidney injury, which then breaks down into LMW-HA moieties, as depicted in Fig. 1 [22]. This transition is indicative of the body’s natural response to injuries, where a pro-inflammatory environment is a necessary step in infection and rapid healing [27, 28]. However, in the case of chronic injury, HMW-HA is needed to promote regenerative rather than fibrotic healing [22].

### Hyaluronan receptors

HA facilitates multifaceted interactions in the damaged tissues by transducing signals via specifically targeted receptors. The dichotomy between the actions of LMW-HA and HMW-HA is partially dictated by the receptors they preferentially bind. LMW-HA tends to bind TLRs 2/4 [29], which are activated by bacterial products and further induce innate immunity by signaling downstream macrophage and dendritic cells [30]. Uncontrolled inflammation with excessive macrophage and dendritic cell activation leads to slow wound closure in chronic wounds [31]. Contrarily, HMW-HA typically binds CD44 and leads to anti-inflammatory reactions to reduce macrophage infiltration and fibrosis [29].

Notably, although CD44 is a primary receptor for HMW-HA, its binding is not exclusive, so LMW-HA signaling of inflammatory pathways can periodically be transduced through the same receptor. This explains the diversity of cellular responses to injury, from inducing an anti-inflammatory environment and promoting immune cell inactivation [32, 33] to causing adverse tissue remodeling [34, 35]. Additionally, alternative RNA splicing and post-translational processing of the CD44 receptor molecule can produce distinct variants that alter the way HA signals and other ligands are transduced [36].

Other receptors that HA binds include the receptor for HA-Mediated Motility (RHAMM), the lymphatic endothelial cell hyaluronic acid receptor 1 (LYVE1), and the hyaluronan receptor for endocytosis (HARE). These receptors are present on various organs and multiple cell types, and HA can accordingly exert differential effects based on the receptors and their located cells. For example, HA binding LYVE1 promotes lymphatic tissue proliferation [37], whereas HA binding HARE in lymph nodes internalizes and clears HA [38].

### Hyaluronan binding proteins

HA is known to crosslink with a variety of proteins that include inter-alpha-trypsin inhibitor (IαI), tenascin, TSG-6, pentraxin, and TSP-1 [9]. IαI facilitates the formation of pericellular matrix around fibroblasts and supports HMW-HA stability [39], both of which are associated with scarless wound healing [19]. Proteoglycans, including biglycan, versican, aggrecan, and neurocan, represent a unique class of ECM molecules that interact with HA to guide development and response to disease [40].

As previously discussed, the effects of HA are multifold as its MW variants and auxiliary protein interactions dictate downstream signaling pathways and disease progression. The following content will focus specifically on mechanisms of HA’s contribution to inflammation and fibrosis in the kidney.

## Hyaluronan in the kidney

Over the past 15 years, HA has been observed to induce varying effects in kidney injury and disease that largely depend on the study model but are considerably influenced by its MW. For example, Ito et al. showed that HMW-HA bound to CD44 promotes proximal tubular cell (PTC) migration *in vitro* via MAPK activation [41]. Although their data support the concept that cellular responses to injury are driven by changes in HA MW variants [42–44], the mechanisms of how HA MW influences kidney injury remain unclear [45]. We will address this gap of knowledge by exploring the multifaceted functions of HA for the pathogenesis of the most common kidney diseases in the following sections (Table 2 summarizes the effects of HMW- and LMW-HA on kidney diseases using the studies discussed in this review).

**Table 2:** **The double-edged effects of HA in kidney diseases**. This table outlines the beneficial and detrimental effects of HMW- and LMW-HA in the various kidney diseases discussed in this review. Here, one can appreciate that HMW-HA lends itself to protective effects, whereas LMW-HA promotes the deleterious characteristics of each disease

Disease	Positive effects of HA	Negative effects of HA
Acute kidney injury (AKI)	IL-10-induced HMW-HA reduces fibrosis in I/R model [22]	LMW-HA [46]-CD44 interaction increases the presence of fibrotic molecules (collagen, α-SMA) and causes tubular damage [47]
Chronic kidney diseases (CKD)	Can potentially serve as a biomarker to distinguish between CKD and AKI in certain clinical cases	Increases pro-fibrotic cells and molecules (macrophage presence, CD44 and LYVE-1 expression, α-SMA levels) [46]
Diabetic nephropathy	Maintains structure of glomerular endothelium [48]; HMW-HA associated with less CD44-dependent inflammation [49]	Elevated levels associated with disease development [50]
IgA nephropathy		HA-CD44 interaction plays a role in disease development [51] and fibrotic complications (crescentic glomerulonephritis) [52]
Obstructive uropathy	IL-10-induced HMW-HA reduces fibrosis [22]	Acts as nidus for calcium stone formation to cause obstructive disease [53–55]
Transplant	Can serve as a predictive biomarker for unsuccessful transplant [56]	Associated with organ rejection [57]
Vesicoureteral reflux	Reduces occurrence of UTIs caused by VUR [58]	

### Potential mechanisms of HA in kidney injury and disease

As a primary HA receptor, CD44 has been extensively modeled in various disease studies where cognate receptor–ligand interactions result in increased scarring. One such study showed that increased CD44 is expressed in the injured kidneys of normal mice, and CD44 KO mice subjected to UUO showed increased tubular injury and apoptosis but decreased renal fibrosis [59]. Consistently, development of fibrotic glomerular disease has been associated with CD44^+^ glomerular cells [60] since cells that promote post-injury healing can be recruited, such as macrophages. Another study found that *Hyal2,* in conjunction with the CD44 isoform CD44v7/8, mediates bone morphogenetic protein-7 (BMP7) and regulates myofibroblast differentiation and its capacity to drive fibrosis [61].

Similarly, TLR2 mediates significant inflammatory responses after kidney injury, as evidenced by ischemia/reperfusion (I/R) injury in a TLR2 knockout mouse model that resulted in a decrease of leukocytes, chemokines, and cytokines at the injury site. In comparison to controls, TLR2 knockout mice had decreased kidney injury. Biglycan, an HA binding protein, can induce inflammation via TLR2/4 to recruit pro-inflammatory Th1 and Th17 subsets, ultimately leading to kidney fibrosis [62–65]. Conversely, a hyaluronidase study showed that a lack of *Hyals 1* and *2* increases inflammation and alpha-smooth muscle actin (a-SMA) in an I/R injury mouse model. This underscores the roles of *Hyals 1* and *2* in preventing the buildup of excessive HA to reduce inflammation and fibrosis [66].

### Acute kidney injury

Acute kidney injury (AKI) continues to increase in prevalence, and with repeated injury, AKI is known to be a significant risk factor for developing chronic kidney disease (CKD) [67].

Hypoxia, an inadequate supply of oxygen to the tissue, is one leading cause of AKI. This condition, usually caused by heart and/or lung diseases, can be modeled by I/R injury in preclinical animal models. Decreased HA expression has been associated with reduced inflammation and enhanced renal recovery in post-I/R injury [47]. While HA has been suggested to promote a pro-inflammatory environment in the I/R model above, the MW variant of HA involved in the progression of the injury remains undefined. However, a similar study used the I/R injury model and found that HMW-HA accumulated one day post-injury but was found to undergo degradation into smaller fragments over time [46]. These findings suggest that LMW-HA is the likely mediator of a pro-inflammatory environment, which leads to AKI. In support of this hypothesis, we have demonstrated that the presence of IL-10-induced HMW-HA after I/R injury is cytoprotective and anti-fibrotic [22].

### Kidney fibrosis and CKD

CKD is particularly impactful to children due to it being a lifelong disease with no cure. Etiology of pediatric CKD is significantly different than in adults, with congenital anomalies of the kidney and urinary tract predominating in children less than 12 years of age, and glomerulonephritis as the primary cause in older children [68].

Repetitive AKI often leads to continued activation of fibroblasts and ultimately to tubulointerstitial fibrosis, an inevitable outcome of CKD. Han et al. reported the increased expression of HA, CD44, and LYVE-1 in areas of fibrotic tissue and noted that HA accumulation was associated with an increase in α-SMA [69], thus creating a pro-inflammatory and fibrogenic milieu in a CKD animal model.

Although the ultimate outcome of CKD is interstitial fibrosis leading to kidney failure, the identification of patients with progressive disease is challenging in the absence of high-risk biomarkers. Given the presumed role of HA as a prominent mediator of fibrosis in CKD patients, HA serum levels could help clinicians provide more assertive diagnoses. Akin et al. showed that the average HA level in sera was substantially increased in CKD patients versus AKI patients. Additionally, HA serum concentration was found to selectively correlate with serum albumin concentration and proteinuria in the CKD group [70]. These findings suggest that HA concentration in serum can serve as a biomarker to distinguish between CKD and AKI in patients with uremia and unknown kidney function.

### Obstructive uropathy

Human obstructive uropathy is a common condition usually caused by kidney stones, infection, blood clots, or tumors. One study found that HA levels varied similarly between obstructed and unobstructed kidneys due to the uninjured kidney compensating for HA requirements left unfulfilled by the damaged kidney. Interstitial HA levels were noted to increase after an obstruction because papillary mesenchymal cells stimulated HA synthesis [71].

**Fig. 2:**
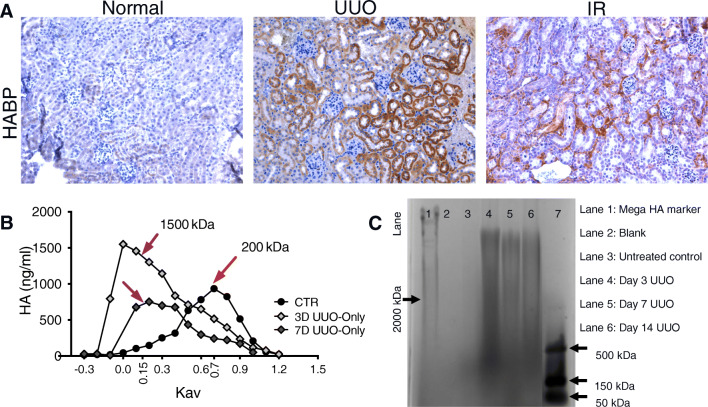
**HA expression and molecular weight changes in normal and diseased mouse kidneys. A.** Images of HA binding protein (HABP) staining of the cortex from control, 7-day UUO kidney and 7-day IR kidney show increased HA accumulation after UUO injury. Scale bars 50 μm. **B.** Plots of total incorporated [3H] in labeled HA samples were used to determine the relative MW of HA synthesized by control and 3D UUO kidneys (*n* ≥ 3 per condition) using Sephacryl S-1000 chromatography. Data were plotted as HA concentration versus the partition coefficient (Kav), showing an increase in HA size distribution in samples with UUO injury. **C.** The extracted HA from control and 3-, 7-, and 14-day untreated and treated UUO kidneys is shown on a 0.5% agarose gel electrophoresis, supporting the chromatography results

**Fig. 3:**
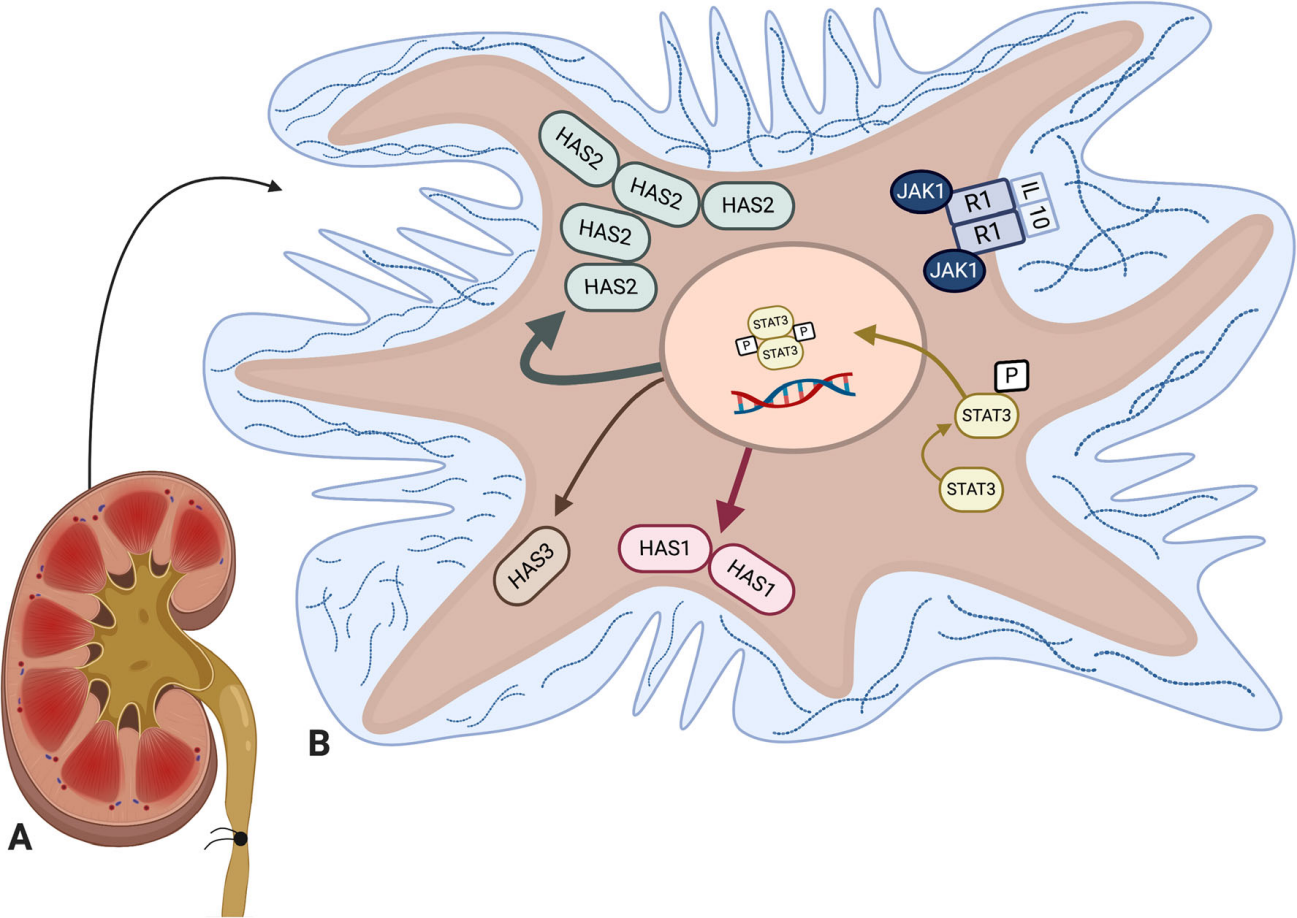
**IL-10 stimulation of renal fibroblasts induces HMW-HA production via STAT3 pathway.** This figure is adapted from our recent publication [22]. It illustrates how IL-10 stimulates renal fibroblasts, activates the STAT3 pathway, and upregulates HA synthesis, especially HMW-HA synthesized by HAS2 which ultimately results in cytoprotection and less fibrosis. Our data demonstrate reduced fibrosis and better-preserved tubules. **A.** Kidney with UUO treated with IL-10. **B.** Fibroblast from renal cortex with HA MW variants in ECM. This schematic is a broad illustration and is not drawn to scale. Created with BioRender.com

Similar to its function in AKI, HMW-HA is beneficial in reducing fibrosis after obstruction [22], indicating that HA MW could play an ameliorating role in the disease. In our recent publication, we show that HMW-HA significantly increases in the interstitium immediately after UUO onset, peaking at 3 days post-induction and indicating an initial pro-regenerative and cytoprotective response to injury. Over time, however, levels of LMW-HA increased—possibly due to uncontrolled inflammation and increased HYALs (Fig. 2)—ultimately resulting in fibrosis. A schematic illustration reveals how HMW-HA could attenuate UUO-induced interstitial fibrosis (Fig. 3).

HA is particularly relevant in obstructive uropathy because it can form gel-like matrices that are negatively charged, granting them the ability to bind crystals. Some data show HA is effective in preventing precipitation of calcium salts in solution, and under physiological conditions, HA can bind multiple calcium carboxyl groups and prevent crystallization [72]. Conversely, other reports on the role of HA in kidney stone formation have shown that it actually serves as a binder for precipitated calcium salts in the renal medulla and cortex, which can eventually lead to Randall’s plaques and nephrocalcinosis [53–55]. Critically, however, none of these studies assessed the molecular weight of the HA responsible for the observed effect on obstructive pathogenesis.

### Diabetic nephropathy

Diabetic nephropathy is the leading cause of CKD 5 worldwide, which can affect patients living with either type I (T1D) or type II (T2D) diabetes mellitus [73, 74]. Although T1D was traditionally associated with children and adolescents and T2D was associated with adults, rising childhood obesity has led to increased incidence of T2D in the youth population [75]. In either case, HA has long been implicated in the development of diabetic nephropathy in several *in vitro* studies, where elevated levels were found in diabetic animal models [50]. While HA has been identified as a mediator of interstitial fibrosis, it does not indicate the actual progression of diabetic nephropathy despite its overexpression in the kidney [73].

One of the pathologic processes leading to diabetic nephropathy is damaged endothelium, where HA is a key constituent of the glycocalyx layer and can thus serve as a more sensitive biomarker for diabetic nephropathy. To identify the effects of endothelial HA loss in diabetic nephropathy, one study found that mice lacking endothelial HAS2 exhibited an abnormal glomerular endothelial structure [48]. Similarly, another study showed decreased HA levels in diabetic rat kidneys at eight weeks [76]. HA has also been tested as a treatment for diabetic nephropathy, since HMW-HA in T2D mice reduced inflammation and glomerulosclerosis [49]. Given that diabetic nephropathy is a progressive disease, earlier detection of the disease by assessment of HA levels could have diagnostic and prognostic benefits.

### IgA nephropathy

IgA nephropathy (IgAN) is characterized by the accumulation of immunoglobulin A (IgA), an antibody that induces inflammation, in the glomeruli of the kidney. In severe cases, this inflammatory process can form irreversible fibrotic crescents that ultimately lead to CKD [77].

In conjunction with HA, which regulates leukocyte activation and migration upon binding to CD44, osteopontin binds to the same receptor to promote cell migration and adhesion. Since HA and OPN interactions with CD44 have been implicated in crescent formation, one group investigated whether such interactions are implicated in the development of IgAN. Their work showed that not only was HA/CD44 binding associated with IgAN, but a correlation existed between the interstitial expression of osteopontin and CD44, and between the extent of tubulointerstitial damage and chronic glomerular lesions [51]. Later, another group studied the role of α-SMA as a crescent formation-associated protein in crescentic glomerulonephritis and found that α-SMA, CD44, HA, and osteopontin levels are upregulated at early stages of the disease. Furthermore, CD44–HA and CD44–osteopontin complexes were found to facilitate cell-matrix and myofibroblast interactions, which likely transduce critical signals in the development of crescents [52].

These collective data suggest that interactions between α-SMA, CD44, HA, and osteopontin play a role in the development of crescentic glomerulonephritis and that targeting HA-related actions could yield therapeutic benefits.

### Vesicoureteral reflux

Vesicoureteral reflux (VUR) is a condition occurring in ~ 1% of the pediatric population in which urine flows retrograde from the bladder into the ureters and eventually into the kidneys [78]. VUR is classified as primary when caused by incomplete closure of valves at the vesicoureteral junction, or as secondary when caused by high pressures in the bladder [79].

HA has been tested as a therapeutic agent for this condition, with multiple clinical studies showing a Dextranomer/Hyaluronic acid (Dx/HA) injection as a safe and effective treatment for primary VUR patients. One study demonstrated that a Dx/HA copolymer decreased VUR-induced urinary tract infections (UTIs) [58], and other similar research showed a near-85% success rate in resolution of VUR with concomitant ureteropelvic junction obstruction [80]. Since VUR also strongly correlates with kidney fibrosis, other work has shown that endoscopic treatment using Dx/HA can be effective in reducing fibrosis [81]. Furthermore, incidence rates of UTIs after open surgery are 2–5× greater than in a Dx/HA treatment, dependent on concurrent febrility [82]. The efficacy of an HA-based therapeutic in VUR highlights its safety profile and sets a precedent for the potential benefits of HA in other kidney diseases.

## Conclusion

Understanding the double-edged nature of HA MW variants enables insight into the seemingly paradoxical role of HA in the body. Even HMW-HA, known to be anti-inflammatory/fibrotic, can be rendered deleterious if broken down to shorter oligosaccharides or LMW variants. These size variants and their corresponding interactions with auxiliary players like HAS, HYAL, and HA receptors and binding proteins implicate HA as part of a highly nuanced signaling pathway in various kidney diseases. In fact, the conversion of HA from HMW to LMW in context of the HAS/HA/CD44 signaling complex has been found to mediate tumor progression and thus presents a promising therapeutic target for kidney cancer research [83]. Additionally, HA can serve as a potential biomarker [56] as upregulation of HA by growth factors and cytokines has been observed in transplant rejection [57]. This results in accumulation of HA in the renal cortex and sclerotic vessels as opposed to its normal presence in the medulla under physiologic conditions [84, 85]. These promising clinical use cases underscore the necessity of further research on HA and its auxiliary molecules, especially in context of its MW. As researchers uncover greater complexities of the HA signaling mechanism, these insights may be leveraged to enhance pediatric patient outcomes across various kidney diseases.

## Glossary

4-MU: 4-methylumbelliferone
CKD 5: stage 5 chronic kidney disease
GAG: glycosaminoglycan
HA: hyaluronic acid/hyaluronan
HARE: HA receptor for endocytosis
HAS: hyaluronan synthase
HMW-HA: high-molecular-weight hyaluronic acid
HYAL: hyaluronidase
LMW-HA: low-molecular-weight hyaluronic acid
LYVE-1: lymphatic vessel endothelial HA receptor-1
MW: molecular weight
RHAMM: receptor for HA-mediated motility
TLR 2/4: toll-like family receptor 2/4
UUO: unilateral ureteral obstruction
